# Phlorizin Prevents Glomerular Hyperfiltration but not Hypertrophy in Diabetic Rats

**DOI:** 10.1155/2008/305403

**Published:** 2008-08-26

**Authors:** Slava Malatiali, Issam Francis, Mario Barac-Nieto

**Affiliations:** ^1^Department of Physiology, Faculty of Medicine, Kuwait University, P.O. Box 24923, Safat 13110, Kuwait; ^2^Department of Pathology, Faculty of Medicine, Kuwait University, P.O. Box 24923, Safat 13110, Kuwait

## Abstract

The relationships of renal and glomerular hypertrophies to development of hyperfiltration and proteinuria early in streptozotocin-induced diabetes were explored. Control, diabetic, phlorizin-treated controls, and diabetic male Fischer rats were used. Phlorizin (an Na^+^-glucose cotransport inhibitor) was given at a dose sufficient to normalize blood glucose. Inulin clearance (C_inulin_) and protein excretion rate (PER) were measured. For morphometry, kidney sections were stained with periodic acid Schiff. At one week, diabetes PER increased 2.8-folds (*P* < .001), C_inulin_ increased 80% (*P* < .01). Kidney wet and dry weights increased 10%–12% (*P* < .05), and glomerular tuft area increased 9.3% (*P* < .001). Phlorizin prevented proteinuria, hyperfiltration, and kidney hypertrophy, but not glomerular hypertrophy. Thus, hyperfiltration, proteinuria, and whole kidney hypertrophy were related to hyperglycemia but not to glomerular growth. Diabetic glomerular hypertrophy constitutes an early event in the progression of glomerular pathology which occurs in the absence of mesangial expansion and persists even after changes in protein excretion and GFR are reversed through glycemic control.

## 1. INTRODUCTION

Nephropathy occurs in about 35% of patients with diabetes mellitus and diabetic nephropathy is responsible for the majority of kidney dialysis and transplants [1]. Renal hyperfiltration and hypertrophy are early manifestations of diabetic nephropathy and much research has been devoted to elaborate if these are major contributors to development of overt renal disease.

Glomerular growth has been seen as the initial renal event in diabetes and found to precede tubular growth [2]. It was believed that glomerular growth with the associated increase in surface area for filtration as observed in diabetic animals and patients [3, 4] is a major determinant of renal hyperfiltration [5].

Others suggest instead that hyperfiltration is the initial event and is thought to occur as a consequence of a decreased afferent arteriolar tone in hyperglycemia, probably mediated by early glycosylation products [6]. Excess filtration then would lead to glomerular hypertension and hypertrophy of glomerular cells in response to excessive stretch [7, 8], resulting eventually in glomerular sclerosis [9].

Recently, it has been proposed that whole kidney growth precedes [10] and leads to hyperfiltration [11, 12], with a major contribution of tubular rather than of glomerular hypertrophy to the development of hyperfiltration through altered tubuloglomerular feedback. It is proposed that in uncontrolled diabetes there is an increased tubular Na^+^-glucose reabsorption [13], due to the increase in filtered glucose load and to increased expression of SGLT1, SGLT2 and GLUT2 transporters in proximal tubule cells [14]. The decreased distal delivery of Na^+^ will cause an increase in GFR through tubuloglomerular feedback (TGF) [12, 15].

Although renal (glomerular and tubular) growth and hyperfiltration may contribute significantly to development of overt diabetic nephropathy [9], the interrelationships between these early renal diabetic events are not clear. Therefore, the aim of this study was to identify the importance of glomerular and renal morphological changes to the development of hyperfiltration and proteinuria in early experimental diabetes, and to study the effect of blocking tubular Na^+^-glucose reabsorption on glomerular and renal hypertrophic growth and on hyperfiltration and proteinuria.

## 2. MATERIALS AND METHODS

### 2.1. Animals

Male Fischer rats (8 weeks old) were placed in a room with an 8:00–20:00 light, 20:00–8:00 dark cycle, kept at 22.3 ± 0.3°C and 31.2 ± 0.8% humidity. The rats had free access to water and standard rat chow (801151, Special Diets Services, UK). All animals were cared for in accordance with the *Guide for the Care and Use of Laboratory Animals* and all experimental protocols used in this study were approved by the Research Administration Committee at Kuwait University. Four groups were used: control rats (C), diabetic rats (D), diabetic rats treated with Phlorizin (DPLZ), and control (nondiabetic) rats treated with phlorizin (CPLZ). This group was added to study any direct renal effects of phlorizin.

### 2.2. Induction of diabetes

Diabetes was induced by intraperitoneal (*i.p*) injection of 55 mg/kg Streptozotocin (STZ, S-0130, Sigma, USA), dissolved in 50 mM trisodium-citrate buffer, pH = 4.5. Controls were given the citrate buffer (vehicle) alone. After STZ injection, animals were placed in metabolic cages and development of diabetes was confirmed 16 hours later if pronounced glucosuria and polyuria developed.

### 2.3. Phlorizin treatment

Diabetic (DPLZ) and control rats (CPLZ) were treated with phlorizin (P-3449, Sigma, USA) starting 16 hours after STZ or citrate buffer *IP* injection. Phlorizin was dissolved at room temperature in propylene glycol (1,2-propanediol, 82282, Fluka, Switzerland). The first day the rats were given a total of 400 mg/kg phlorizin *s.c.* split into doses of 200 mg/kg at 8:00 AM and at 8:00 PM. The second day the dose was raised to 400 mg/kg twice daily and the treatment was continued for 6 days [16].

Blood glucose levels were measured twice daily in the treatment group on samples taken from the tail, using a glucometer (GLUCOTREND 2, Roche, Germany). Nonfasting blood glucose levels were measured in all groups before sacrifice, 7 days after treatment.

### 2.4. Protein excretion rate

Urinary protein concentration was measured using a modified Lowry assay [17]. Protein excretion rate (PER) was calculated from urinary protein concentration (Up, mg/ml) and urine flow rate (*V*, ml/24hrs). The urine samples were collected over twenty four hours from rats placed in metabolic cages.

To assess tubular proteinuria, the urinary excretion of beta_2_-microglobulin was tested. The concentration of beta_2_-microglobulin in rat urine was measured using beta_2_-Microglobulin PET Kit (K 0052, DAKO, Denmark). Changes in absorbance read at 340 nm (Hitachi model 911, Boehringer Mannheim, Germany) were proportional to the concentration of beta_2_-microglobulin in standards of known concentrations. The urinary excretion rate of beta_2_-microglobulin was calculated from its urinary concentration and the urine flow rate.

### 2.5. Renal hemodynamics

Glomerular filtration rate was estimated by measuring the renal plasma clearance of inulin (C_inulin_). Each rat was weighed and anaesthetized with Inactin (Thiobutabarbital sodium C_10_H_15_N_2_NaO_2_S, T-133, Sigma, UK) at a dose of 100 mg/kg IP. The left femoral artery was catheterized for continuous monitoring of arterial blood pressure by a pressure transducer. Blood samples were collected from the arterial catheter. Tracheostomy was performed to insure proper ventilation. The femoral vein was catheterized and an infusion with sterile Ringer's solution (154 mM NaCl, 5.61 mM KCl, 2.16 mM CaCl_2_, 5.95 mM NaHCO_3_, 5.55 mM glucose) was started at a rate of 0.06 ml/min using a syringe pump (Harvard Apparatus Ltd, UK). The bladder was cannulated with a stainless steel cannula for urine collection. The cannula was connected to a flexible tube of the appropriate size for urine collection. For estimation of urine flow rate, the tube was carefully removed, the urine emptied in a tared Eppendorf tube and weighed.

After a 45–60 mininutes period of stabilization, an infusion containing inulin (from Chicory root, I-2255, Sigma, USA) was started. The perfusion solution contained 36 mg/mL of inulin in Ringer's, and was infused at a rate of 0.06 mL/min. Priming dose of 160 mg/kg was given intravenously. After a 30 minutes equilibration period, three or four timed samples of urine were collected (15–20 minutes each). At the midpoint of each urine collection period, an arterial blood sample of 300 *μ*l was collected in dry heparinzed tubes, and centrifuged at 2000 g for 10 minutes. Plasma and urine samples were stored at −70°C for later analysis of inulin concentrations.

Concentration of inulin was measured in urine and plasma samples [18] after precipitation with 10% TCA. Clearance values were expressed in mL/min per 100 g of initial body weight.

### 2.6. Morphometry

Seven days after STZ or vehicle treatment, rats were anesthetized; the right kidney was removed, weighed, dried, and reweighed. The left kidney was perfusion-fixed with 10% neutral-phosphate buffered formalin. After 15 minutes perfusion, the left kidney was removed and a 3 mm thick transverse cross-section was cut and placed in formalin. Twenty four hours later sections were embedded in wax, cut into 4 micron sections, and mounted on APES (3-aminopropyltriethoxysilane, Sigma, A-3648) coated slides for morphological studies. Periodic acid-Schiff (PAS) stain was used for measurements of total glomerular tuft and mesangial matrix areas [19]. The sections were hydrated and then placed in 0.5% periodic acid (5–10 minutes). The slides were washed, successively dehydrated in 70%, 95%, 100% ethanol, and 100% xylene, protected with a cover slip using DPX, and allowed to dry overnight. A total of 15 to 20 completely round glomeruli showing their tuft attached at the hilum were selected randomly from all renal cortical zones in each animal. Morphometry of glomeruli was done with a CAS 200 cell analysis system (Becton & Dickinson Image Cytometry Systems, USA). Total glomerular tuft area (GA, mm^2^) was calculated after manual tracing of each tuft contour. Glomerular tuft volume GTV was estimated from

$$\text{GTV}\;=\;b/k\times (\text{GA}{)}^{3/2}[20],$$

where *b* = 1.38 is a shape coefficient for a sphere and *k* = 1.1 a size distribution coefficient.

Mesangial matrix area in each glomerular tuft was measured using a threshold method that selectively highlights all PAS-positive areas within the tuft.

### 2.7. Statistical analysis

All results were expressed as mean ±SEM and were analyzed by one way ANOVA to establish differences between groups. When *F* was significant, comparisons between any two groups were further tested using unpaired two-tailed students *t*-test for equal or unequal variances according to the equality of variance test. Correlation studies were done using Pearson's test. The software used was SPSS 11 for Windows. For all statistical tests, a *P* value of less than 0.05 was considered significant.

## 3. RESULTS

### 3.1. Blood glucose, body, and kidney weights, urine flow and protein excretion rates in phlorizin-treated and untreated diabetic rats

Streptozotocin-induced diabetes caused significant increases in blood glucose concentration, urine flow rate, and PER and a significant decrease in body weight (Table 1).

**Figure 1:**
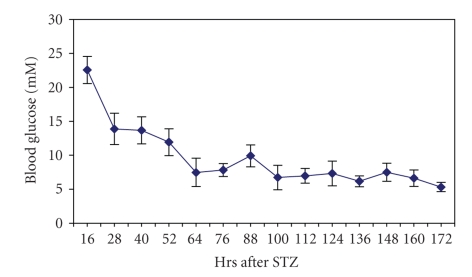
Blood glucose concentrations in phlorizin-treated diabetic rats (*n* = 5).

Seven-day treatment of diabetic rats with phlorizin reduced the blood glucose levels to normal values (Table 1) within 60 hours (Figure 1); however, there was still significant diuresis and decrease in body weight (Table 1). Diuresis and body weight loss were also observed in phlorizin-treated control rats (Table 1).

One week of diabetes led to small (10–12%) but significant (*P* < .05) increases in the kidney wet and dry weights expressed as percentages of initial body weights (Table 1). Phlorizin treatment prevented the mild renal growth observed in diabetic rats. Phlorizin had no effect on renal wet or dry kidney weights in control rats.

### 3.2. Glomerular morphology and proteinuria in phlorizin-treated and untreated diabetic rats

**Figure 2:**
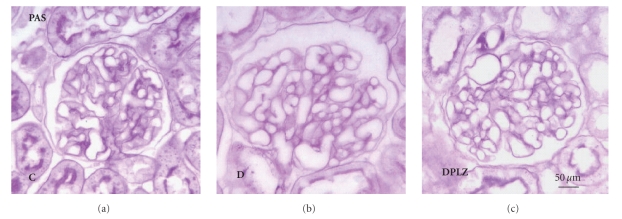
Glomerular morphological changes due to one-week diabetes in Fischer rats. Glomerular tuft area significantly increased after one week of diabetes. PAS positive area exhibited no change in one-week diabetics (D) when compared to controls (C). Phlorizin treatment (DPLZ) did not prevent glomerular growth. (4 *μ*m, paraffin sections, periodic acid-Schiff (PAS) stain, ×400).

At one-week diabetes, glomerular capillary tuft area and volume are increased by 9.3% and 14.6%, respectively, with no significant change in PAS-positive mesangial matrix area (Table 2, Figure 2). Phlorizin treatment did not prevent glomerular growth (Figure 2, Table 2) nor altered mesangial matrix area. However, phlorizin prevented diabetic proteinuria since PER in PLZ-treated diabetic rats was not significantly different from that in PLZ-treated controls. The 1.7 fold higher PER observed in PLZ-treated control rats compared with untreated controls is consistent with an independent effect of phlorizin on the renal handling of proteins.

Excretion of beta_2_-microglobulin was measured to assess tubular protein reabsorption [20]. There were no traces of beta_2_-microglobulin in urine of control, and diabetic rats. However, there were significant amounts of beta_2_-microglobulin in the urine of phlorizin-treated control and diabetic rats. Urinary excretions of beta_2_-microglobulin were 0.018 ± 0.01 and 0.023 ± 0.01 mg/24h in PLZ-treated controls and PLZ-treated diabetic rats, respectively (*P* = 0.7). Thus, while one-week diabetes does not alter the renal handling of beta_2_-microglobulin, phlorizin has an inhibitory effect on the tubular reabsorption of beta_2_-microglobulin in control and diabetic rats.

**Figure 3:**
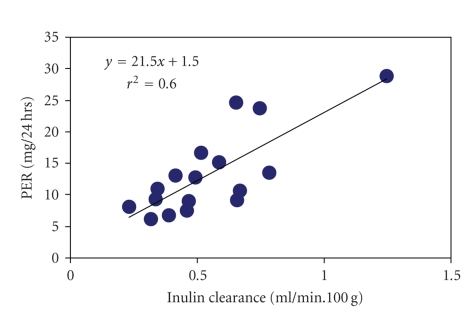
Correlation of protein excretion rate (PER) with inulin clearance (*P* < .001) in control, diabetic, and phlorizin-treated control and diabetic Fischer rats. Inulin clearance was normalized to 100 g of initial body weight.

The early 3-4 fold diabetes-associated increase in PER was not found to correlate with glomerular tuft or mesangial matrix areas or volumes, but instead was correlated with C_inulin_ (Figure 3, *r* ^2^ = 0.6, *P* < .01), suggesting that is mostly related to functional rather than to gross morphological glomerular changes.

### 3.3. Glomerular hemodynamic changes in phlorizin-treated and untreated diabetic rats

Mean arterial blood pressure was similar in all the groups studied (Table 2). One-week diabetic rats had an 80% higher (*P* < .01) C_inulin_ than vehicle-treated nondiabetic time controls (Table 2). Phlorizin treatment prevented the diabetes-associated increase in C_inulin_. Phlorizin had no significant hemodynamic effect in control rats.

**Figure 4:**
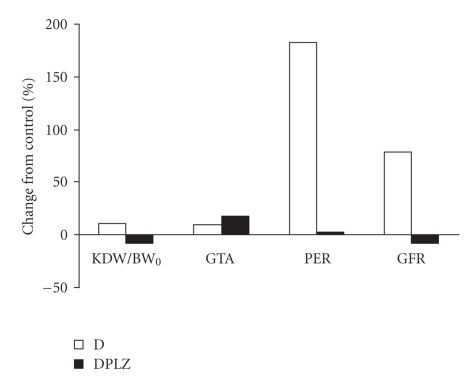
Percent changes from control of kidney dry weight to initial body weight ratio (KDW/BW_0_), glomerular tuft area (GTA), protein excretion rate (PER), and glomerular filtration rate (GFR) in diabetic (D) and phlorizin-treated diabetic (DPLZ) rats. Phlorizin treatment prevented renal growth, proteinuria and hyperfiltration, but did not prevent glomerular hypertrophy in diabetic rats. Absolute values and statistics are presented in Tables 1, 2, and 3.

The effects of phlorizin on diabetic-induced changes are summarized in Figure 4. These results show that in diabetes, phlorizin, an inhibitor of proximal tubule sodium-glucose reabsorption, prevents glomerular hyperfiltration, proteinuria and whole kidney (tubular) growth but not glomerular tuft growth.

## 4. DISCUSSION

One week after induction of streptozotocin diabetes, renal and glomerular hypertrophy and hyperfiltration were observed in Fischer rats. Renal hypertrophy, although small, was significant and occurred independently of body weight changes. Hyperfiltration was evident by a significant increase in C_inulin_ similar to that reported earlier in SD rats [21].

One week of phlorizin treatment prevented renal whole kidney growth and hyperfiltration; however, it did not prevent glomerular tuft growth. The sustained glomerular growth with phlorizin treatment is probably related to the fact that hyperglycemia persisted for at least 60 hours after PLZ and STZ treatments. Hyperglycemia induces the release of growth factors such as VEGF [22, 23], TGF-*β* [24], and Angiotensin II [25] by resident glomerular cells and by infiltrating macrophages [26] that invade the glomeruli as early as one day after STZ treatment [26]. Once induced, glomerular growth persists even when glycemia was normalized with PLZ for at least three days, which indicates that glomerular growth can persist independently of the rates of Na^+^-glucose cotransport, and of hyperglycemia. Trophic effects of phlorizin per se on the glomerulus have not been reported. Podocytes [27] and glomerular mesangial cells [28] show SGLT 1 expression indicating that Na^+^-glucose cotransport is active in these cells. Blocking this pathway with phlorizin will reduce glucose entry [28] and decrease their size [29] rather than induce their growth or proliferation.

By contrast, diabetic renal tubular growth, manifested by increases in total wet and dry kidney weights [30], was totally prevented by phlorizin treatment. This indicates that in STZ diabetes, hypertrophy of renal tubular cells depends on hyperglycemia and the associated increase in tubular Na^+^-glucose cotransport, and is rapidly reversed by normalizing blood glucose concentration. Thus, diabetic renal tubular and glomerular hypertrophies show different dynamics upon normalization of blood glucose and probably involve different growth pathways.

The diabetes-associated increase in GFR detected in this study is independent of glomerular growth, since with phlorizin treatment GFR was not elevated despite sustained glomerular growth. Furthermore in diabetes, while the increase in GFR was of 80%, that of the glomerular tuft cross-sectional area was only 14%. Since the flow is proportional to the square of the cross-sectional area, most of the increase in GFR was likely due to changes in pre-or postglomerular resistances rather than in glomerular cross-sectional area.

Similar to the findings in this study, phlorizin had no hemodynamic effects in control rats [31]. In control rats, Na^+^-glucose cotransport represents at most 5% of total Na^+^ transport in the nephron [31]. However, in diabetic rats, proximal Na^+^-dependent glucose reabsorption increases [13, 32] with increases in filtered glucose load and in expression of glucose transporters (GLUT 2) [14, 32]. Enhanced proximal reabsorption decreases distal sodium delivery leading to preglomerular dilatation and hyperfiltration through tubuloglomerular feedback (TGF) [12]. Acute infusion of phlorizin, at a dose that does not normalize the blood glucose level, was shown to decrease GFR [13]. Blocking the diabetes-enhanced sodium glucose cotransport with PLZ decreases expression of GLUT2 [33], greatly decreases proximal reabsorption of sodium, increases its distal delivery, and leads to normalization in GFR, possibly through reversal of TGF [12]. The effect of phlorizin in preventing hyperfiltration in the current study was similar to that reported earlier [13] but occurred with a higher PLZ dose which resulted in normoglycemia and a normal filtered glucose load within 60 hours of PLZ treatment. Therefore, in our experiments, the effect of phlorizin on GFR could be direct, due to inhibition of tubular reabsorption of glucose and indirect, through normalization of the blood glucose level and of processes such as enhanced proximal sodium-glucose cotransport that are dependent on hyperglycemia. The prevention of hyperfiltration with either low or high doses of PLZ suggests that activation of TGF is the main mechanism by which hyperglycemia increases GFR in diabetes and that its other effects on renal vessels have little influence on early diabetic hyperfiltration.

The reduction in C_inulin_ with PLZ cannot be accounted for by an effect of PLZ on extracellular fluid volume (ECFV). Phlorizin-treated diabetic rats had twenty-four-hour urine flow rate and diuretic response to Ringer's infusion similar to diabetic rats indicating similar state of hydration in both groups. Therefore, PLZ had no significant effect on the ECFV of these animals. Phlorizin treatment reduced body weight in both DPLZ and CPLZ rats, but reduced C_inulin_ only in DPLZ animals. Thus, the change in body weight associated with PLZ treatment has no direct effect on C_inulin_.

Hyperfiltration was correlated with mild proteinuria in this model of early diabetes. However, since a correlation does not necessarily indicate a causal relationship, the current data do not allow us to define whether diabetes leads to mild proteinuria only through hyperfiltration or if additional effects are involved. Early diabetic proteinuria was totally prevented by one week phlorizin treatment indicating that it, as well as hyperfiltration, is due to processes dependent on hyperglycemia and independent of glomerular tuft growth which was not reversed when glycemia was normalized with phlorizin treatment. The absence of excretion of beta_2_-microglobulin, a marker of tubular protein transport [20], observed in diabetic rats indicates that there is no early effect of diabetes on tubular protein reabsorption.

In this study we observed persistent glomerular hypertrophy in the absence of proteinuria, hyperfiltration, or whole kidney growth when the glycemia was controlled. We speculate that persistent glomerular growth may be a precursor of glomerular sclerosis and of renal insufficiency (low GRF) that occur in the absence of proteinuria or of kidney hypertrophy in about 20% of patients with diabetic nephropathy [34–36].

## 5. CONCLUSION

This study shows that early in diabetes renal tubular and glomerular hypertrophy, mild proteinuria and hyperfiltration occur in Fischer rats. The data indicate that the renal functional changes observed early in diabetes (hyperfiltration and proteinuria) cannot be accounted for by the observed glomerular hypertrophy and, in contrast to glomerular growth, are readily reversed by phlorizin-induced normalization of blood glucose. Glomerular hypertrophy is an early event in the development of STZ-diabetes-induced glomerular pathology, occurred in the absence of mesangial matrix expansion and persisted after short-term normalization of the glycemia. The alleviation of early renal functional and some structural (tubule hypertrophy) changes in diabetes with phlorizin, as it does in the retina [29], supports its potential therapeutic role in minimizing diabetic microvascular disease. Further studies are needed to investigate if diabetes-induced glomerular hypertrophy leads to glomerulosclerosis and how it can be prevented or reversed.

## Figures and Tables

**Table 1:** Body and kidney weights, blood and urinary changes in Fischer rats at one-week diabetes. Bwt_0_ = Initial body weight, Bwt_*f*_ = final body weight, BG = blood glucose, *V* = urine flow rate measured over 24 hours from rats placed in metabolic cages, PER = protein excretion rate, Wt = weight, C = control, D = diabetic, DPLZ = phlorizin-treated diabetic, CPLZ = phlorizin-treated controls. Results are expressed as mean ± SE and compared using unpaired two-tailed student's *t*-test.

	Bwt_0_	Bwt_*f*_	BG	*V*	PER	Kidney wet	Kidney dry
	(g)	(g)	(mM/l)	(ml/24hrs)	(mg/24hrs)	wt/Bwt_0_%	wt/Bwt_0_%
C	196.5 ± 3.2	208 ± 6.5	5.6 ± 0.3	6.5 ± 1.04	8.2 ± 1.5	0.39 ± 0.01	0.085 ± 0.003
*n* = 14							

D	192 ± 2.0	177.8 ± 4.9**	27.7 ± 1.4^*##*^	63.4 ± 6.4^*##*^	23.2 ± 5.6^*#*^	0.43 ± 0.01^*#*^	0.095 ± 0.001^*#*^
*n* = 15							

DPLZ	199.6 ± 4.2	175.7 ± 2.6**	5.9 ± 1.1	47.5 ± 4^*##*^	14.3 ± 2.6^*#*^	0.39 ± 0.02	0.078 ± 0.005
*n* = 9							

CPLZ	200 ± 2.7	187.6 ± 4.2*^φ^	5.1 ± 0.2	21.3 ± 2.6^*##φφφ*^	14 ± 1.8^*#*^	0.4 ± 0.02	0.082 ± 0.004
*n* = 5							

* = *P* < .05, ** = *P* < .001 compared to initial weight using paired two-tailed student's *t*-test. ^*#*^ = *P* < .05, ^*##*^ = *P* < .001 compared to control, ^*φ*^ = *P* < .05, ^*φφφ*^ = *P* < .001 CPLZ *versus* DPLZ.

**Table 2:** Glomerular morphometry in Fischer rats at one-week diabetes: *n* = number of rats, *N* = total number of observations, GTV = glomerular tuft volume. Tuft A = glomerular tuft area, MMA = mesangial matrix area. All variables were analyzed using one way ANOVA, and then between group differences were compared using unpaired two-tailed student's *t*-test.

	Control	Diabetic	DPLZ
	*n* = 5, *N* = 72	*n* = 6, *N* = 89	*n* = 5, *N* = 70
GTV	486 ± 11	557 ± 11***	621 ± 153***
*μ*m^3^ × 10^3^			

Tuft A	5293.9 ± 117.8	5796.7 ± 74.4***	6225.1 ± 102.9***
*μ*m^2^			

MMA	163.3 ± 6	159.1 ± 4.5	164.6 ± 6.6
*μ*m^2^			

MMA/tuft *A*%	3.07 ± 0.1	2.77 ± 0.1	2.65 ± 0.1**

** = *P* < .01, *** = *P* < .001, compared to control.

**Table 3:** Inulin clearances (GFR) in the experimental groups: C = control, D = diabetic, DPLZ = phlorizin-treated diabetic rats, CPLZ = phlorizin-treated controls. C_inulin_ = clearance of inulin, Bwt_0_ = initial body weight, *V* = urine flow rate during clearance experiment, MBP = mean blood pressure. Clearance values were normalized to 100 g of initial body weight. Results are expressed as mean ±SEM and compared using student's *t*-test.

	Control (*n* = 7)	Diabetic (*n* = 5)	DPLZ (*n* = 5)	CPLZ (*n* = 5)
C_inulin_ (ml*·*min^−1^ *·*100 g^−1^)	0.47 ± 0.02	0.84 ± 0.1**	0.43 ± 0.05^*#*^	0.61 ± 0.07
Bwt_0_ (g)	203.2 ± 5.6	196.2 ± 1.5	200.2 ± 3.8	200 ± 2.7
*V* (*μ*l*·*min^−1^ *·*100 g^−1^)	5.6 ± 0.5	9.6 ± 1.3*	13. ± 3.1*	6 ± 0.7
MBP (mmHg)	126.6 ± 3.3	123.9 ± 4.2	121.8 ± 2.2	123.0 ± 2.3

* = *P* < .05, ** = *P* < .01 compared to control. ^*#*^ = *P* < .01 compared to diabetic.
